# Social and Behavioral Factors Associated With Diabetes in Southern California vs the US

**DOI:** 10.1001/jamanetworkopen.2025.38377

**Published:** 2025-10-22

**Authors:** Alexandra Descarpentrie, Sevan Esaian, Ben Allen, Juan Espinoza, Vishal Midya, Nathan Young, Tanya L. Alderete, Michael I. Goran

**Affiliations:** 1 Department of Pediatrics, The Saban Research Institute, Children’s Hospital Los Angeles, University of Southern California; 2 Department of Psychology, University of Kansas, Lawrence; 3 Stanley Manne Children’s Research Institute, Ann and Robert H. Lurie Children’s Hospital of Chicago, Chicago, Illinois; 4 Department of Pediatrics, Northwestern University Feinberg School of Medicine, Chicago, Illinois; 5 Department of Environmental Medicine and Public Health, Icahn School of Medicine at Mount Sinai, New York; 6 Department of Environmental Health and Engineering, Johns Hopkins Bloomberg School of Public Health, Baltimore, Maryland

## Abstract

**Importance:**

Diabetes disproportionately impacts communities across the US and in Southern California, where there is a large Hispanic population. Diabetes prevalence continues to increase, and more studies are needed to understand behaviors and socioecological factors contributing to diabetes risk regionally and nationally.

**Objective:**

To identify social and behavioral correlates (SBC) of diabetes in Southern California and to contrast these findings with national data to help ensure that public health strategies are appropriately targeted and regionally responsive.

**Design, setting, and participants:**

This ecological and cross-sectional study used modeled estimates from census tracts in Southern California and nationwide, drawing from the Centers for Disease Control and Prevention’s 2024 Population Level Analysis and Community Estimates (PLACES) dataset.

**Exposures:**

Based on PLACES data availability and prior evidence, 24 SBC were selected and were grouped into health outcomes and conditions, prevention, health risk behaviors, health-related social needs, and broader social determinants of health.

**Main Outcomes and Measures:**

Diagnosed diabetes prevalence, also from the PLACES dataset, represented the percentage of adults ever diagnosed with diabetes by a health care professional, excluding gestational diabetes. A total of 24 indicators were tested for their association with diabetes prevalence using a gradient-boosted regression tree method.

**Results:**

In Southern California (5420 census tracts composed of approximately 18.5 million adults), diagnosed diabetes had a mean (SE) prevalence of 11.29% (0.06), ranging from 1.4% (0.05) to 33.6% (1.37), compared with 11.52% (0.02) nationally (62 480 tracts composed of approximately 253 million adults). In Southern California, leveraging an explainable machine learning approach, 5 key correlates were identified accounting for 67% of the estimate in the model: physical inactivity (31%), routine check-ups (14%), binge drinking (11%), lack of insurance (6%), and food insecurity (5%). While some key correlates overlapped regionally and nationally, their estimated contributions differed. Additionally, obesity, receipt of food stamps, being aged 65 years or older, and persons of ethnic or racial minority background (all except White, non-Hispanic) were key correlates nationally but not in Southern California.

**Conclusion:**

In this cross-sectional study, key correlates of diagnosed diabetes in Southern California included physical inactivity, access to health care, and food insecurity, while nationally, obesity, food stamp participation, older age (≥65 years), and racial or ethnic minority status were also key. These findings point to possible regional differences in the factors linked to diabetes prevalence and highlight the need for further research to determine their significance and potential for guiding targeted interventions.

## Key Points

**Question:**

What are the key social and behavioral factors associated with diagnosed diabetes in Southern California, and how do they differ from those observed at the national level?

**Findings:**

This cross-sectional study of 5420 census tracts composed of approximately 18.5 million adults in Southern California identified physical inactivity, binge drinking, routine check-ups, lack of insurance, and food insecurity as key factors associated with diagnosed diabetes. While some factors overlapped nationally, obesity, food stamp participation, persons aged 65 years and older, and persons of racial or ethnic minority background were key factors nationwide but not regionally.

**Meaning:**

These findings point to possible regional differences in the factors associated with diabetes prevalence and highlight the need for further research to determine their significance and potential for guiding targeted interventions.

## Teaser

This cross-sectional study evaluates social and behavioral factors associated with diabetes in Southern California and contrasts these findings with national data.

## Introduction

In 2021, 38.4 million people (11.6% of the US population) had diabetes, including 2 million with type 1 and 8.7 million undiagnosed cases.^1^ Type 2 diabetes prevalence is highest among American Indian and Alaskan Native (13.6%), Black (12.1%), Hispanic (11.7%), and Asian American (9.1%) populations.^1,2^ Given that Hispanic people make up 45% of Southern California’s population, mostly of Mexican origin due to the region’s large border with Mexico, followed by Central Americans,^3,4^ targeted efforts are essential in this area to effectively address the community’s distinct health needs.

Research on risk factors and diabetes prevention has shown that, alongside individual health behaviors, important contributing factors include economic, social, and policy-related conditions, as well as limited health care access.^5,6,7^ Resource-deprived environments (characterized by poverty, low education, and limited health care) play a key role in diabetes prevalence and outcomes. While these social and behavioral correlates (SBC) have been studied individually, few studies have examined their collective impact, underscoring the need for data-driven approaches.^7^ Machine learning (ML) can handle complex, interrelated factors, but its lack of explainability limits its practical use in health research. Explainable ML addresses this by identifying key factors and refining our understanding of health outcomes.^8,9^

Previous studies^4^ have linked community-level indices (eg, Healthy Places Index, CalEnviroScreen, and Social Vulnerability Index) to diabetes prevalence in Southern California. However, these indices aggregate multiple social and environmental factors, making it difficult to assess their individual contributions. Additionally, the COVID-19 pandemic disrupted health behaviors and health care access with lasting effects.^5,7,10^ These changes highlight the need for updated insights into diabetes correlates.

This exploratory and ecological study, designed as a hypothesis-generating analysis, used a place-based, data-driven approach with 2024 Population Level Analysis and Community Estimates (PLACES) modeled data from the US Centers for Disease Control and Prevention (CDC) to examine SBC associated with diagnosed diabetes in Southern California. Our objectives were to (1) identify key SBC of diabetes prevalence in the region, and (2) compare these findings with national trends to highlight regional differences that may be obscured in national-level analyses.

## Methods

### Data Source and Study Population

The 2024 CDC PLACES release provides estimates for 52 measures in counties, incorporated and census-designated places, census tracts, and zip code tabulation areas across the US.^11,12^ PLACES measures are grouped into 7 sections (eFigure 1 in Supplement 1): (1) health outcomes and conditions, (2) prevention, (3) health risk behaviors, (4) disabilities, (5) health status, (6) health-related social needs, and (7) social determinants of health (SDOH). We retained the original PLACES classification for consistency. However, conceptually, we considered SDOH to encompass not only section 7 (broader structural social factors) but also elements from sections 2 and 6 (more immediate social-related factors linked to health). Most PLACES estimates (sections 1-6) were derived using small-area estimation (SAE) methods^13^ based on individual behavioral- and health-related responses from the 2022 Behavioral Risk Factor Surveillance System (BRFSS)^14^ and American Community Survey data (ACS) (2018-2022).^15^ In contrast, the 9 SDOH estimates (section 7) were sourced from the 2020 CDC/Agency for Toxic Substances and Disease Registry Social Vulnerability Index (SVI),^16^ which assesses social vulnerability at the county and census tract levels using only the ACS data to reflect broader and structural social factors. All PLACES estimates are accompanied by their corresponding CIs.

This study did not require institutional review board approval or informed consent as it used only publicly available, deidentified census tract–level data. Reporting follows the Strengthening the Reporting of Observational Studies in Epidemiology (STROBE) reporting guidelines.

### Outcome

The outcome measure was diagnosed diabetes prevalence, obtained from the PLACES Health Outcomes/Conditions section. This reflects the percentage of adults having ever been told by a physician, nurse, or other health professional that they have diabetes other than diabetes during pregnancy.

### Tested Correlates

Our variable selection approach was intentional and informed by expert judgment. We selected 24 indicators from the 52 PLACES variables that were relevant for estimating diagnosed diabetes prevalence based on previous studies examining these associations.^2,5,7,17,18^ This selection prioritized potential upstream correlates of diabetes while avoiding variables likely to reflect downstream complications. eFigure 1 in Supplement 1 provides a detailed description of all PLACES variables and the correlates selected.

### Statistical Analysis

eFigure 2 in Supplement 1 outlines the analytical approach used in this analysis. Only complete cases were included in this analysis. For objective 1, we analyzed 5420 Southern California census tracts. For objective 2, we repeated the analysis at the national level with 62 480 tracts.

We used extreme gradient boosting (XGBoost), a gradient-boosted regression tree method, to estimate diagnosed diabetes prevalence.^19^ As a nonparametric approach, this model handles multicollinearity and captures nonlinearities and higher-order interactions without requiring data transformations. This makes it ideal for exploring the multifactorial correlates of diabetes, where the effect of individual variables may depend on complex interactions with others. All variables were expressed as percentages. Data were split into training (3795 census tracts [70%] for Southern California and 43 738 census tracts [30%] for the US) and test (1625 census tracts [30%] in Southern California and 18 742 census tracts [70%] in the US) sets, ensuring balanced diabetes distribution (eTable 1 in Supplement 1). Although estimation was not the primary goal, this splitting strategy was used to assess model stability within the population studied. To train the model, we implemented cross-validation with 10 folds using spatial blocks to account for spatial autocorrelation,^20^ recognizing that nearby census tracts often share similar characteristics. The optimal combination of hyperparameters was selected based on performance using smaller root mean squared error (RMSE), which measures the average magnitude of estimation errors (eTable 2 in Supplement 1). RMSE is particularly useful because it penalizes larger errors more heavily, helping ensure overall accuracy. Final model evaluation used *R*^2^ on the independent test set.

To interpret the model, we applied Shapley additive explanations (SHAP).^21^ SHAP assigns a unique value to each correlate for every observation (in this case, census tract), indicating how much that variable’s value influences the model’s estimation relative to the average estimation across the dataset, while accounting for all other variables and interactions. Positive SHAP values indicate the variable increased diabetes prevalence estimations, while negative values suggest a decrease. Since the outcome is in percentage points, SHAP values follow the same scale. We used normalized mean absolute SHAP values to evaluate each variable’s estimated contribution, identifying an SBC as key if it accounted for at least 5% of the total estimated contribution.

To explore how the type of variables may influence the observed associations, we built separate models using (1) proximal or intermediate variables only (eg, health behaviors, health outcomes, prevention, health-related social needs), and (2) distal or structural variables only (eg, SDOH as defined by PLACES). To test model robustness, we also repartitioned the dataset using different commonly used train-test splits (75/25 and 80/20), and conducted a bootstrap-based sensitivity analysis (50 bootstraps) incorporating SEs for each variable and census tract to account for feature variability. We then averaged the SHAP values across these bootstraps to determine whether the key factors identified in the main analysis remained consistent. Finally, to test the assumption of spatial autocorrelation, we conducted a standard (nonspatial) k-fold (k = 10) cross-validation. The same workflow from the main analysis (eFigure 2 in Supplement 1) was applied to sensitivity analyses. All the previously mentioned sensitivity analyses were conducted exclusively on the Southern California dataset.

All analyses were conducted in R version 4.3.3 (R Project for Statistical Computing), with spatial cross-validation implemented through the blockCV package.^20^ SHAP values and corresponding plots were generated using the kernelshap and shapviz packages.

## Results

### Descriptive Statistics

**Figure 1.**
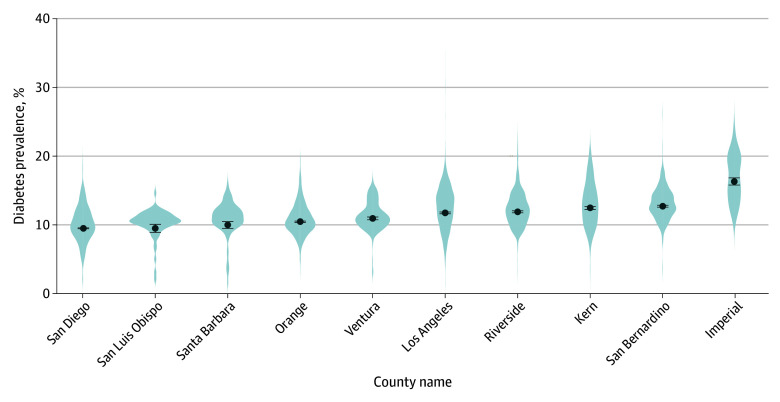
Percentage of Diagnosed Diabetes per County in Southern California (n = 5420 Census Tracts); Population Level Analysis and Community Estimates, 2024 Dots represent the mean values for each county and error bars indicate SEs.

Mean (SE) diagnosed diabetes prevalence across all census tracts in Southern California (5420 census tracts composed of approximately 18.5 million adults) was 11.29% (0.06), while mean (SE) diagnosed diabetes prevalence across all census tracts in the US was 11.52% (0.02). Figure 1 displays a violin plot and eFigure 3 in Supplement 1 displays a map of the observed diagnosed diabetes prevalence in Southern California broken down by county. The prevalence of diagnosed diabetes in Southern California also varied across counties, with Imperial County having the highest at 16.3% (SE, 0.52), while San Diego (9.4% [SE, 0.13]) and San Luis Obispo (9.4% [SE, 0.56]) had the lowest prevalences. Notably, diabetes prevalence ranged from 1.4% (SE, 0.05) to 33.6% (SE, 1.37) across census tracts, with 37 tracts (68%) exceeding 20%. Nationally, diagnosed diabetes prevalence ranged from 1.1% (SE, 0.10) to 37.8% (SE, 1.42) across census tracts, with 2567 tracts (4.1%) exceeding 20%. Distributions for each of the 24 potential correlates for Southern California are shown in the Table. eTable 1 in Supplement 1 presents those distributions for the training and testing sets. Among the 24 correlates, 26 correlation pairs (9.4%) showed correlations above 0.9, and 149 correlation pairs (54.0%) were moderate or lower (≤0.6) (eFigure 4 in Supplement 1). None correlated with diabetes above 0.9, only 1 exceeded 0.8, and 11 correlation pairs (45.8%) were 0.6 or less (eTable 3 in Supplement 1). eTable 4 in Supplement 1 displays a table showing the observed correlate distributions in the US.

**Table.** Population Level Analysis and Community Estimates Variable Characteristics in Southern California (n = 5420 Census Tracts)

Characteristic	Mean of percentages (SE)^a^
Health outcomes	
Depression	20.95 (0.04)
Obesity	27.49 (0.08)
Prevention	
Routine checkups	71.5 (0.05)
Lack of health insurance	8.35 (0.07)
Health risk behaviors	
Short sleep duration	35.93 (0.05)
Binge drinking	17.78 (0.04)
Current cigarette smoking	11.20 (0.05)
No leisure-time physical activity	22.68 (0.10)
Health-related social needs	
Food insecurity	14.89 (0.12)
Feeling socially isolated	34.86 (0.05)
Housing insecurity	13.81 (0.10)
Receipt of food stamps	13.30 (0.13)
Utility services shut-off threat	6.44 (0.05)
Lack of social and emotional support	31.54 (0.07)
Lack of reliable transportation	8.91 (0.06)
Social determinants of health	
Persons aged ≥65 y	11.98 (0.15)
No broadband internet subscription among households	6.00 (0.07)
Crowding among housing units	5.29 (0.09)
Housing cost burden among households	31.39 (0.20)
No high school diploma	11.26 (0.19)
Persons living below 150% of the poverty level	16.56 (0.23)
Persons of racial or ethnic minority status	68.35 (0.50)
Single-parent households	4.13 (0.06)
Unemployment	4.99 (0.06)

^a^ Weighted descriptive statistics (means and standard errors) described the census tracts in the analytic sample with weights applied to adjust for estimate precision; larger areas had more reliable estimates. Weights were calculated as the inverse of the standard error for each estimate.

### Main Analysis

#### Model Performance and Key Correlates of Diagnosed Diabetes Prevalence in Southern California

**Figure 2.**
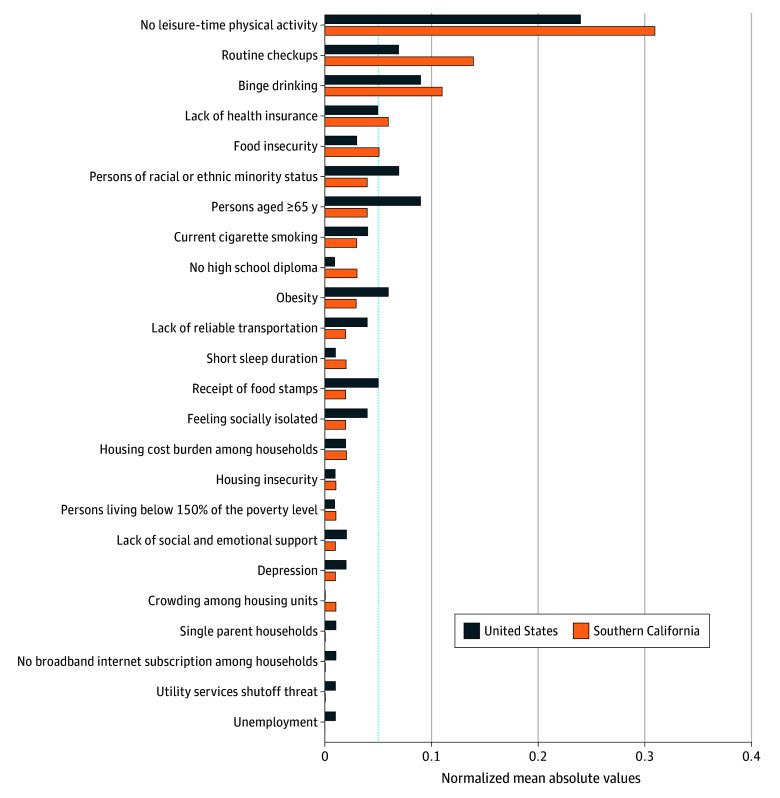
Key Correlates of Diagnosed Diabetes: Shapley Additive Explanations–Based Ranking in Southern California and National Models The bar chart displays the normalized mean absolute Shapley additive explanations (SHAP) values for the 24 correlates, comparing their relative contributions with estimated diabetes prevalence in Southern California and the US. Variables are ordered in descending importance based on the Southern California model. Values shown as 0 represent contributions below 0.01. A dotted vertical line marks the 0.05 threshold for visual reference. SHAP assigns a unique value to each correlate for every observation (in this case, census tract), indicating how much that variable’s value influences the model’s estimation relative to the average estimation across the dataset while accounting for all other variables and interactions. We used normalized mean absolute SHAP values to evaluate each variable’s contribution, identifying a social and behavioral covariate as key if it accounted for at least 5% of the total estimated contribution.

Our model explained 96% of the between-tract variance in diagnosed diabetes prevalence in Southern California (eFigure 5 in Supplement 1). Five key correlates accounted for 67% of the estimated contribution: (1) no leisure-time physical activity (31%), (2) routine check-up (14%), (3) binge drinking (11%), (4) lack of health insurance (6%), and (5) food insecurity (5%) (Figure 2).

**Figure 3.**
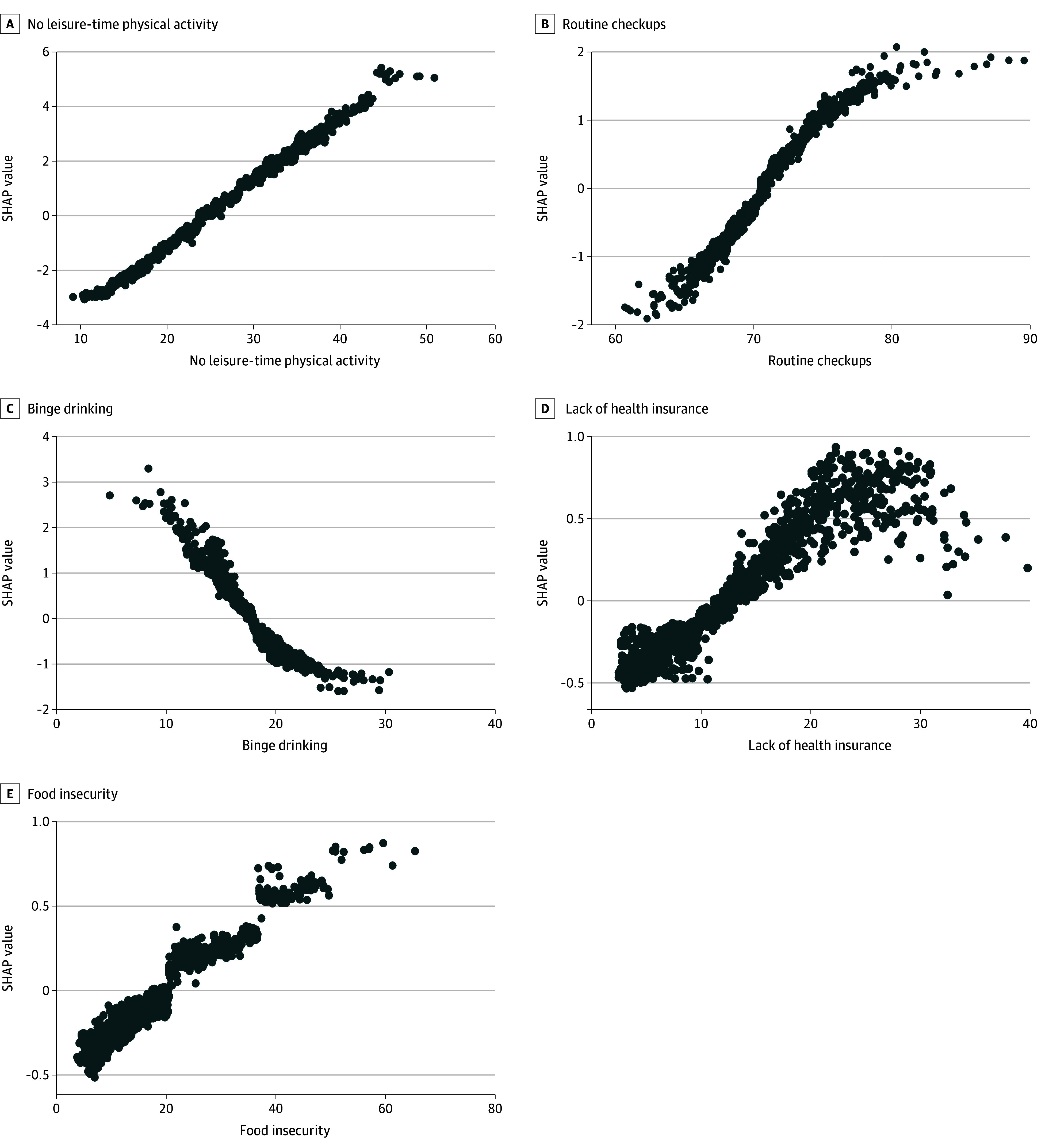
Dependence Plots for Associations Between Key Correlates and Shapley Additive Explanations Values, Southern California. These plots display the association between each key correlate (estimated contribution explained ≥5%) and the model’s estimated diagnosed diabetes prevalence, while accounting for complex, nonlinear interactions learned by the algorithm. Each point represents 1 census tract from the test set. The vertical axis shows the Shapley additive explanations (SHAP) value, which reflects the contribution of the variable (on the x-axis) to the model’s estimation for that tract. These plots are intended to offer a nuanced view of how each key correlate is associated with model outputs across its range of values. SHAP values above 0 reflect estimations of diabetes prevalence higher than the mean in percentage points; SHAP values below 0 reflect estimations of diabetes prevalence below the mean in percentage points. The x-axis unit is percentage.

Dependence plots (Figure 3) showed that higher estimated diabetes prevalence was overall associated with increased rates of no leisure-time physical activity, routine checkups, lack of health insurance, and food insecurity. Conversely, lower binge drinking rates were overall associated with higher estimated prevalence. While these associations were overall positively or inversely associated with estimated diagnosed diabetes prevalence, they depicted clear nonlinearities, with inflection points around 40% for no leisure-time physical activity, 50% for food insecurity, and 10% for lack of health insurance, while the effects of routine checkups, and binge drinking plateaued at 75% and 20%, respectively.

#### National vs Regional Model Performance and Differences in Key Correlates

At the national level, the model’s performance almost mirrored that of Southern California, explaining 95% of the variance in diabetes prevalence. The key variables included 8 SBC, explaining 72% of the estimated contribution, compared with 5 key correlates in Southern California. While the top variables were fairly similar to those identified in Southern California (Figure 2), their relative importance varied. Additionally, at the national level, obesity, receipt of food stamps, persons aged 65 years or older, and persons of racial or ethnic minority background (except white, non-Hispanic) emerged as important contributors, while none of these were identified as key correlates in Southern California.

### Sensitivity Analyses

When using only distal or structural variables, our model explained 75.3% of the between-tract variance in diagnosed diabetes prevalence. Four key correlates accounted for 86% of the estimated contribution: (1) persons of racial or ethnic minority status (25%), (2) persons aged 65 years or older (24%), (3) no high school diploma (21%), and (4) persons living below 150% of the poverty level (16%) (eTable 5 in Supplement 1). With only proximal or intermediate variables, the model explained 95.5% of the variance. The top 5 factors contributed 82% of the estimated contribution: (1) no leisure-time physical activity (30%), (2) having routine checkups (14%), (3) lack of health insurance (12%), (4) food insecurity (10%), (5) binge drinking (9%), and (6) feeling socially isolated (7%) (eTable 6 in Supplement 1).

The models generated using different data splitting ratios (80/20 and 75/25) respectively explained 93.8% and 96.0% of the between-tract variance in diagnosed diabetes prevalence. Out of the 5 key correlates identified in the main analysis in Southern California, 4 were consistently found when using these 2 different splits, with the fifth one (food insecurity) being identified in only 1 of them (eTables 7 and 8 in Supplement 1). The results obtained averaging SHAP values over 50 bootstraps closely aligned with the results from the main analysis, confirming the stability of key correlates (eTable 9 in Supplement 1). Of note, the 95% bootstrap CIs for all SHAP values exclude 0, indicating that each variable significantly contributed to the estimations of diagnosed diabetes. Finally, k-fold cross-validation confirmed the robustness of our results, with a similar variance explained (95.9%) and consistent correlate rankings (eTable 10 in Supplement 1).

## Discussion

Our ecological analysis identified key SBC of diagnosed diabetes prevalence across the region of Southern California, where addressing rising rates is a priority. No leisure-time physical activity was the top correlate, followed by routine check-ups, binge drinking, lack of insurance, and food insecurity. Key correlates and their contributions differed nationally, with obesity, receipt of food stamps, persons aged 65 years or older, and persons of racial or ethnic minority background being correlates at the national level but not in Southern California.

While past studies linked some SBC to diabetes, the key correlates and their associations in our study differ from earlier findings based on regression^22^ and ML models that used earlier, less complete releases of the PLACES data. Notably, a previous study^23^ conducted in New York City using the 2020 PLACES release did not include behavioral factors, and thus identified broader socioeconomic correlates such as low education and high SNAP participation. However, our results suggest that including these variables may not have altered the findings, as key correlates vary regionally. Indeed, nationally, 8 major SBC emerged, vs 5 in Southern California.

Physical inactivity was the strongest correlate of diabetes, showing a nonlinear association with prevalence, independent of various socioeconomic indicators. Regular exercise improves glucose uptake and insulin sensitivity,^24^ as well as obesity,^25^ and may therefore function as a protective factor. While the obesity-diabetes link is well established, our findings suggest this association may either be weaker in Southern California and/or more dominated by a link with physical inactivity. This result may alternatively reflect the limitations of BMI as a proxy for adiposity compared with measures like waist circumference or may reflect residual confounding.

A higher rate of routine check-ups was a stronger diabetes correlate in Southern California than nationally, underscoring the importance of early detection through screenings like HbA_1c_.^26^ Despite accounting for routine check-ups in our model, lack of health insurance also importantly contributed to higher diabetes prevalence. A possible explanation is that insured individuals are more likely to engage in any other form of preventive health measures.

Alcohol consumption showed counterintuitive results; however, if the PLACES definition of heavy drinking (≥5 drinks for men or ≥4 drinks for women on ≥1 occasion in the past 30 days) more closely reflects moderate intake in practice, then previous studies suggest it may lower diabetes risk by improving insulin sensitivity.^27,28^ While we lacked precise alcohol intake data, future research is needed to explore this further, considering its known associated health risks.^29^

Food insecurity emerged as one of the most important factors in our models, even when accounting for other indicators of economic hardship. This suggests that food insecurity may worsen diabetes through nutritional, behavioral, and mental health pathways, leading to poor diet, insulin resistance, and trade-offs between food, medication, and care. Additionally, food insecurity can lead to mental health issues, such as anxiety, which are risk factors for diabetes.^30^ These mechanisms are broadly relevant, but regional socioeconomic differences likely explain food insecurity’s weaker national association with diabetes.

This study points to potential avenues for improving diabetes outcomes in Southern California. Future prospective studies may further explore the relevance of key factors identified here, such as physical inactivity, missed check-ups, alcohol use, lack of insurance, and food insecurity. If these associations are confirmed, strategies such as promoting accessible fitness programs and optimal food options, walking groups, and ensuring safe outdoor spaces for exercise, along with expanding preventive health care services and insurance coverage, could prove beneficial.

Future research should examine alcohol’s link to diabetes while considering its known liver and heart risks.^29^ On a separate note, exploring the roles of social disorganization and discrimination (variables absent in the current dataset) could offer critical insights into how these social correlates shape the diagnosis of diabetes. Finally, it is essential to investigate how SBC influence patient and practitioner perspectives on diabetes management.

### Limitations and Strengths

Our study has several limitations. Due to the ecological, cross-sectional design, the correlates that we identified indicate contextual associations rather than causal links, with potential for reverse causation. XGBoost captures complex patterns missed by traditional methods, making SHAP a powerful tool for interpreting variable importance. However, SHAP rankings require a shift from regression thinking and remain model-dependent and thus are prone to residual confounding. SHAP may also assign high importance to correlated features due to how XGBoost handles splits (eg, selecting one variable from a correlated group). In Southern California, 3 key correlates are strongly correlated (see eFigure 6 in Supplement 1), though stronger correlations with nonkey variables lessen this concern (see eFigure 7 in Supplement 1). Still, these factors represent distinct public health levers. Additionally, the outcome (self-reported diagnosed diabetes) was not medically confirmed and was itself modeled, which may introduce further uncertainty and biased conclusions. Using prevalence rather than incidence data prevents establishing temporal relationships, making it unclear whether specific SBC are associated with new diabetes cases or with existing cases. We were also unable to distinguish between type 1 and type 2 diabetes or account for undiagnosed cases. Both the outcome and SBC were self-reported, introducing potential misreporting bias in addition to estimation errors from modeled data. Still, SHAP values averaged over 50 bootstraps confirmed the robustness and significance of key correlates. While estimation was not the goal, the strong model performance should be interpreted cautiously, as both outcome and some correlates (from sections 1-6) were modeled from BRFSS data using overlapping variables.^12^ However, moderate correlations between diagnosed diabetes and BRFSS-based correlates reduce concerns of circularity. To limit overfitting, we further applied a 70/30 spatially stratified train-test split. Result consistency, even without spatial constraints, supports robustness.

Despite these limitations, the place-based insights from this study suggest potential regional variation in the correlates of diabetes prevalence. Applying similar models to other regions or datasets may yield valuable context-specific findings and help determine whether observed differences are meaningful, why they arise, and how they could inform more targeted prevention strategies and future research.

## Conclusions

This cross-sectional and ecological study identified key SBC associated with diagnosed diabetes in Southern California. Physical inactivity emerged as the strongest correlate, followed by routine check-ups, binge drinking, lack of health insurance, and food insecurity. While there was some overlap, distinct factors were also identified as key correlates at the national level. These findings suggest potential regional variation in the correlates of diabetes prevalence and underscore the need for further research to understand whether these differences are meaningful, why they exist, and whether they may inform more targeted interventions.

## Notes

Supplement 1. eFigure 1. PLACES Variables, 2024 Release eFigure 2. Analysis Strategy eTable 1. PLACES Variable Characteristics in Southern California for the Training and Test Data Sets (n=5420 Census Tracts)1; PLACES, 2024 eTable 2. Hyperparameters for Model Training eFigure 3. Maps of Observed Diagnosed Diabetes Prevalence Within Southern California by County (n=5420); PLACES, 2024 eFigure 4. Correlation Heatmap of the Considered PLACES Correlates in Southern California (Whole Data Set, n=5420 Census Tracts) eTable 3. Correlations Between the Considered PLACES Correlates and Diagnosed Diabetes in Southern California (Whole Data Set, n=5420 Census Tracts) eFigure 5. Correlations Between the Key Correlates of Diagnosed Diabetes in Southern California (Whole Data Set, n=5420 Census Tracts) eFigure 6. Correlations (Spearman >0.93) Between the Considered Correlates of Diagnosed Diabetes in Southern California (Whole Data Set, n=5420 Census Tracts) eTable 4. PLACES Variable Characteristics in the US (n= 62 480 Census Tracts); PLACES, 2024 eFigure 7. Observed vs. Estimated (% of Diagnosed Diabetes) Plot in Test Data Set (n=1625 Census Tracts in Southern California); PLACES, 2024 eTable 5. Raw and Normalized Mean Absolute Shapley Additive Explanations Values for Test Dataset With Objective Community-Level Variables Only (n=1625 Census Tracts in Southern California); PLACES, 2024 eTable 6. Raw and Normalized Mean Absolute Shapley Additive Explanations Values for Test Dataset With Individual-Level Variables Only (n=1625 Census Tracts in Southern California); PLACES, 2024 eTable 7. Raw and Normalized Mean Absolute Shapley Additive Explanations Values For Test Dataset With a Different Splitting Strategy—80/20 (n=1083 Census Tracts in Southern California); PLACES, 2024 eTable 8. Raw and Normalized Mean Absolute Shapley Additive Explanations Values for Test Dataset With a Different Splitting Strategy—75/25 (n=1,354 Census Tracts in Southern California); PLACES, 2024 eTable 9. Raw and Normalized Mean Absolute Shapley Additive Explanations Values for Test Dataset, Taking Into Account Uncertainty in Analyzed Variables (n=1625 Census Tracts in Southern California); PLACES, 2024 eTable 10. Raw and Normalized Mean Absolute Shapley Additive Explanations Values for Test Dataset, Using K-Fold (k=10) Cross-Validation (n=1625 Census Tracts in Southern California); PLACES, 2024

Supplement 2. Data Sharing Statement
