# Supplementary material for: Social and Behavioral Factors Associated With Diabetes in Southern California vs the US
Source: JAMA Netw Open. 2025 Oct 22;8(10):e2538377. doi: 10.1001/jamanetworkopen.2025.38377 (PMC12547587; doi:10.1001/jamanetworkopen.2025.38377)
Supplement: Supplement 1. — eFigure 1. PLACES Variables, 2024 Release eFigure 2. Analysis Strategy eTable 1. PLACES Variable Characteristics in Southern California for the Training and Test Data Sets (n=5420 Census Tracts)1; PLACES, 2024 eTable 2. Hyperparameters for Model Training eFigure 3. Maps of Observed Diagnosed Diabetes Prevalence Within Southern California by County (n=5420); PLACES, 2024 eFigure 4. Correlation Heatmap of the Considered PLACES Correlates in Southern California (Whole Data Set, n=5420 Census Tracts) eTable 3. Correlations Between the Considered PLACES Correlates and Diagnosed Diabetes in Southern California (Whole Data Set, n=5420 Census Tracts) eFigure 5. Correlations Between the Key Correlates of Diagnosed Diabetes in Southern California (Whole Data Set, n=5420 Census Tracts) eFigure 6. Correlations (Spearman >0.93) Between the Considered Correlates of Diagnosed Diabetes in Southern California (Whole Data Set, n=5420 Census Tracts) eTable 4. PLACES Variable Characteristics in the US (n= 62 480 Census Tracts); PLACES, 2024 eFigure 7. Observed vs. Estimated (% of Diagnosed Diabetes) Plot in Test Data Set (n=1625 Census Tracts in Southern California); PLACES, 2024 eTable 5. Raw and Normalized Mean Absolute Shapley Additive Explanations Values for Test Dataset With Objective Community-Level Variables Only (n=1625 Census Tracts in Southern California); PLACES, 2024 eTable 6. Raw and Normalized Mean Absolute Shapley Additive Explanations Values for Test Dataset With Individual-Level Variables Only (n=1625 Census Tracts in Southern California); PLACES, 2024 eTable 7. Raw and Normalized Mean Absolute Shapley Additive Explanations Values For Test Dataset With a Different Splitting Strategy—80/20 (n=1083 Census Tracts in Southern California); PLACES, 2024 eTable 8. Raw and Normalized Mean Absolute Shapley Additive Explanations Values for Test Dataset With a Different Splitting Strategy—75/25 (n=1,354 Census Tracts in Southern California); PLACES, 2024 eTable 9. Raw and Normali [file jamanetwopen-e2538377-s001.pdf]

Supplemental Online Content

Descarpentrie A, Esaian S, Allen B, et al. Social and behavioral correlates of diabetes in southern California vs the US. *JAMA Netw Open*. 2025;8(10):e2538377. doi:10.1001/jamanetworkopen.2025.38377

eFigure 1. PLACES Variables, 2024 Release

eFigure 2. Analysis Strategy

eTable 1. PLACES Variable Characteristics in Southern California for the Training and Test Data Sets (n=5420 Census Tracts)[1]; PLACES, 2024

eTable 2. Hyperparameters for Model Training

eFigure 3. Maps of Observed Diagnosed Diabetes Prevalence Within Southern California by County (n=5420); PLACES, 2024

eFigure 4. Correlation Heatmap of the Considered PLACES Correlates in Southern California (Whole Data Set, n=5420 Census Tracts)

eTable 3. Correlations Between the Considered PLACES Correlates and Diagnosed Diabetes in Southern California (Whole Data Set, n=5420 Census Tracts)

eFigure 5. Observed vs Estimated (% of Diagnosed Diabetes) Plot in Test Data Set (n=1625 Census Tracts in Southern California); PLACES, 2024

eFigure 6. Correlations Between the Key Correlates of Diagnosed Diabetes in Southern California (Whole Data Set, n=5420 Census Tracts)

eFigure 7. Correlations (Spearman >0.93) Between the Considered Correlates of Diagnosed Diabetes in Southern California (Whole Data Set, n=5420 Census Tracts)

eTable 4. PLACES Variable Characteristics in the US (n= 62 480 Census Tracts); PLACES, 2024

eTable 5. Raw and Normalized Mean Absolute Shapley Additive Explanations Values for Test Dataset With Objective Community-Level Variables Only (n=1625 Census Tracts in Southern California); PLACES, 2024

© 2025 Descarpentrie A et al. *JAMA Network Open*.

eTable 6. Raw and Normalized Mean Absolute Shapley Additive Explanations Values for Test Dataset With Individual-Level Variables Only (n=1625 Census Tracts in Southern California); PLACES, 2024

eTable 7. Raw and Normalized Mean Absolute Shapley Additive Explanations Values For Test Dataset With a Different Splitting Strategy—80/20 (n=1083 Census Tracts in Southern California); PLACES, 2024

eTable 8. Raw and Normalized Mean Absolute Shapley Additive Explanations Values for Test Dataset With a Different Splitting Strategy—75/25 (n=1,354 Census Tracts in Southern California); PLACES, 2024

eTable 9. Raw and Normalized Mean Absolute Shapley Additive Explanations Values for Test Dataset, Taking Into Account Uncertainty in Analyzed Variables (n=1625 Census Tracts in Southern California); PLACES, 2024

eTable 10. Raw and Normalized Mean Absolute Shapley Additive Explanations Values for Test Dataset, Using K-Fold (k=10) Cross-Validation (n=1625 Census Tracts in Southern California); PLACES, 2024

This supplemental material has been provided by the authors to give readers additional information about their work.

© 2025 Descarpentrie A et al. *JAMA Network Open*.

eFigure 1. PLACES Variables, 2024 Release

Health Outcomes (13 measures)

- Arthritis
- Current asthma
- High blood pressure
- Cancer (non-skin) or melanoma
- High cholesterol
- Chronic kidney disease
- Chronic obstructive pulmonary disease
- Coronary heart disease
- *Depression*
- Diagnosed diabetes
- *Obesity*
- All teeth lost among adults aged ≥65 y
- Stroke

Prevention (9 measures)

- *Lack of health insurance*
- *Routine checkup*
- Visited dentist/dental clinic
- Taking medicine to control high blood pressure
- Cholesterol screening
- Mammography use
- Cervical cancer screening
- Colorectal cancer screening
- Adults 65+ up to date on core preventive services, by sex

Health Risk Behaviors (4 measures)

- *Binge drinking*
- *Current cigarette smoking*
- *No leisure-time physical activity*
- *Short sleep duration*

Health Status (3 measures)

- Frequent mental distress
- Frequent physical distress
- Fair or poor self-rated health status

Disabilities (7 measures)

- Hearing disability
- Vision disability
- Cognitive disability
- Mobility disability
- Self-care disability
- Independent living disability
- Any disability

Health Related Social Needs (7 measures)

- *Feelings of social isolation*
- *Receipt of food stamps*
- *Food insecurity*
- *Housing insecurity*
- *Utility services threat*
- *Lack of reliable transportation*
- *Lack of social and emotional suppor*

Social Determinants of Health (9 measures)

- *Persons aged ≥ 65 years*
- *No broadband internet subscription among households*
- *Crowding among housing units*
- *Housing cost burden among households*
- *No high school diploma*
- *Persons living below 150% of the poverty level*
- *Persons of racial or ethnic minority status*
- *Single-parent households*
- *Unemployment*

The social and behavioral correlates selected for the analysis are shown in italics and underlined.

© 2025 Descarpentrie A et al. *JAMA Network Open*.

The following variables were selected for the analysis. This selection prioritized potential upstream correlates of diagnosed diabetes while avoiding variables likely to reflect downstream complications.

1) Health Outcomes/Conditions: Among 13 "semi-objective" health indicators, depression and obesity were included in this study. Though potentially bidirectional, these factors were included based on evidence linking them to diabetes risk[1,2]. Kidney, heart, lung diseases, cancer, and hypertension were excluded as likely diabetes complications.

2) Prevention: We selected two indicators from this section: the percentage of adults aged 18–64 without current health insurance coverage, and the percentage of adults having visited a doctor for a routine checkup. Cancer screening and dental visit variables were excluded as they were deemed conceptually unrelated to diabetes prevalence.

3) Health Risk Behaviors: This data includes estimated prevalence of four adult behaviors that we included: binge drinking (≥5 drinks men/≥4 drinks women in past 30 days), smoking (≥100 cigarettes lifetime and current smoking), physical inactivity (no exercise), and short sleep duration (<7 hours/day).

4) and 5) Disabilities and Health Status (mostly perceived health): We excluded any variables from these sections (representing 10 variables), as they may represent complications or perceptions of having diabetes, rather than correlates.

6) Health-Related Social Needs: This section includes seven key factors, expressed as percentages, such as food and housing insecurity, receipt of food stamps, transportation barriers, social isolation, utility threat, and lack of social and emotional support. We included all these factors in the analysis.

7) Social Determinants of Health: This section includes nine key measures from the SVI, including the percentage of persons aged ≥65 years, households without broadband internet, housing cost burden and crowding, adults aged ≥25 years without a high school diploma, individuals of racial or ethnic minority status (all except white, non-Hispanic; ie, Hispanic or Latino (of any race); Black and African American, Not Hispanic or Latino; American Indian and Alaska Native, Not Hispanic or Latino; Asian, Not Hispanic or Latino; Native Hawaiian and Other Pacific Islander, Not Hispanic or Latino; Two or More Races, Not Hispanic or Latino; Other Races, Not Hispanic or Latino), single-parent households, unemployment, and poverty. All these measures were considered for inclusion.

© 2025 Descarpentrie A et al. *JAMA Network Open*.

eFigure 2. Analysis Strategy

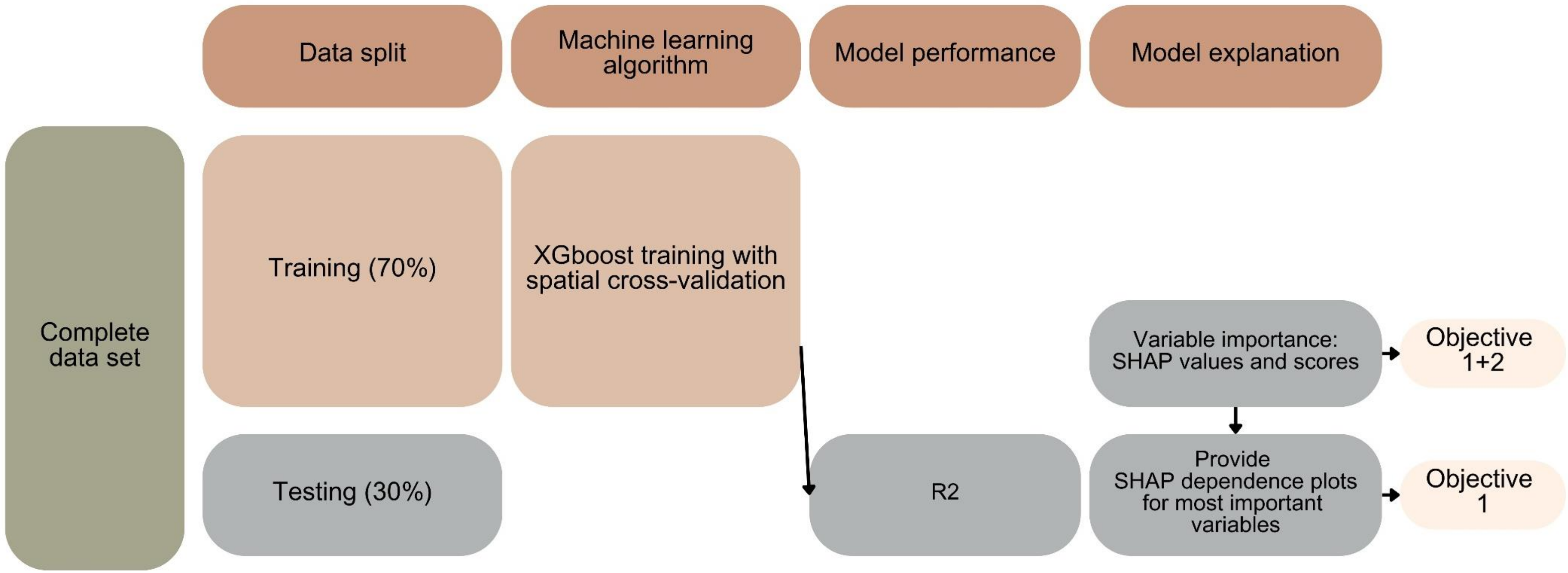

RMSE: root mean square error; R2: R-squared; SHAP: Shapley Additive Explanations; XGBoost: eXtreme Gradient Boost

© 2025 Descarpentrie A et al. *JAMA Network Open*.

eTable 1. PLACES Variable Characteristics in Southern California for the Training and Test Data Sets (n=5420 Census Tracts)[1]; PLACES, 2024

	Mean of percentages (SE[1])	
	**Training set**	**Testing set**
**Health Outcomes**		
Diagnosed diabetes	11.29 (0.07)	11.29 (0.1)
Depression	20.94 (0.04)	20.97 (0.07)
Obesity	27.5 (0.1)	27.45 (0.15)
**Prevention**		
Routine checkups	71.5 (0.06)	71.51 (0.1)
Lack of health insurance	8.34 (0.09)	8.36 (0.13)
**Health-Risk Behaviors**		
Short sleep duration	35.94 (0.06)	35.89 (0.1)
Binge drinking	17.78 (0.05)	17.77 (0.08)
Current cigarette smoking	11.22 (0.06)	11.16 (0.09)
No leisure-time physical activity	22.7 (0.12)	22.62 (0.19)
**Health-Related Social Needs**		
Food insecurity	14.89 (0.15)	14.91 (0.23)
Feeling socially isolated	34.84 (0.06)	34.91 (0.09)
Housing insecurity	13.8 (0.12)	13.83 (0.18)
Receipt of food stamps	13.28 (0.15)	13.33 (0.23)
Utility services shut-off threat	6.44 (0.05)	6.44 (0.08)
Lack of social and emotional support	31.54 (0.09)	31.56 (0.14)
Lack of reliable transportation	8.9 (0.07)	8.93 (0.11)
**Social Determinants of Health**		
Persons aged ≥ 65 years	11.88 (0.19)	12.2 (0.21)
No broadband internet subscription among households	6.01 (0.08)	5.97 (0.12)
Crowding among housing units	5.31 (0.11)	5.24 (0.16)
Housing cost burden among households	31.37 (0.24)	31.45 (0.37)
No high school diploma	11.28 (0.23)	11.22 (0.35)
Persons living below 150% of the poverty level	16.57 (0.27)	16.52 (0.42)
Persons of racial or ethnic minority status	68.6 (0.61)	67.74 (0.88)
Single-parent households	4.1 (0.07)	4.19 (0.1)
Unemployment	4.98 (0.06)	5.04 (0.14)

[1]standard error

Weighted descriptive statistics (means and standard errors) described the census tracts in the analytic sample, with weights applied to adjust for estimate precision, larger areas had more reliable estimates. Weights were calculated as the inverse of the standard error for each estimate.

© 2025 Descarpentrie A et al. *JAMA Network Open*.

eTable 2. Hyperparameters for Model Training

This table presents the hyperparameters used in the model training process. These hyperparameters were determined to enhance estimated accuracy and generalizability.

Hyperparameters	Best value (Southern California and United States)
Nrounds	92
Eta	0.09
Colsample bytree	0.49
Max depth	8
Gamma	2.06
Min child weight	18
Subsample	0.7

Nrounds determines the number of boosting iterations, while **eta** (learning rate) controls how much each tree contributes to the final model. **Colsample bytree** regulates the fraction of features used per tree, promoting diversity, and **max depth** sets the complexity of individual trees. **Gamma** prevents overfitting by requiring a minimum reduction in loss for a split, whereas **min child weight** ensures that leaves contain a sufficient number of observations. Lastly, **subsample** dictates the proportion of training data used for each tree, helping to reduce variance and improve generalization.

© 2025 Descarpentrie A et al. *JAMA Network Open*.

eFigure 3. Maps of Observed Diagnosed Diabetes Prevalence Within Southern California by County (n=5420); PLACES, 2024

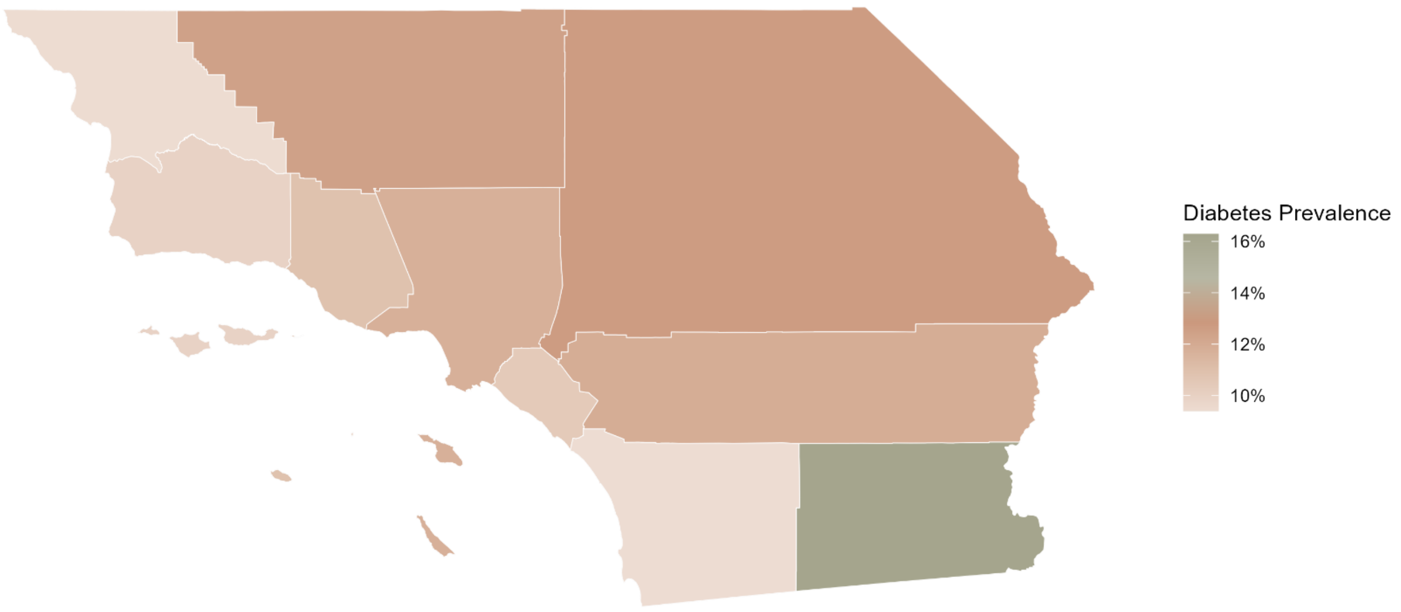

© 2025 Descarpentrie A et al. *JAMA Network Open*.

eFigure 4. Correlation Heatmap of the Considered PLACES Correlates in Southern California (Whole Data Set, n=5420 Census Tracts)

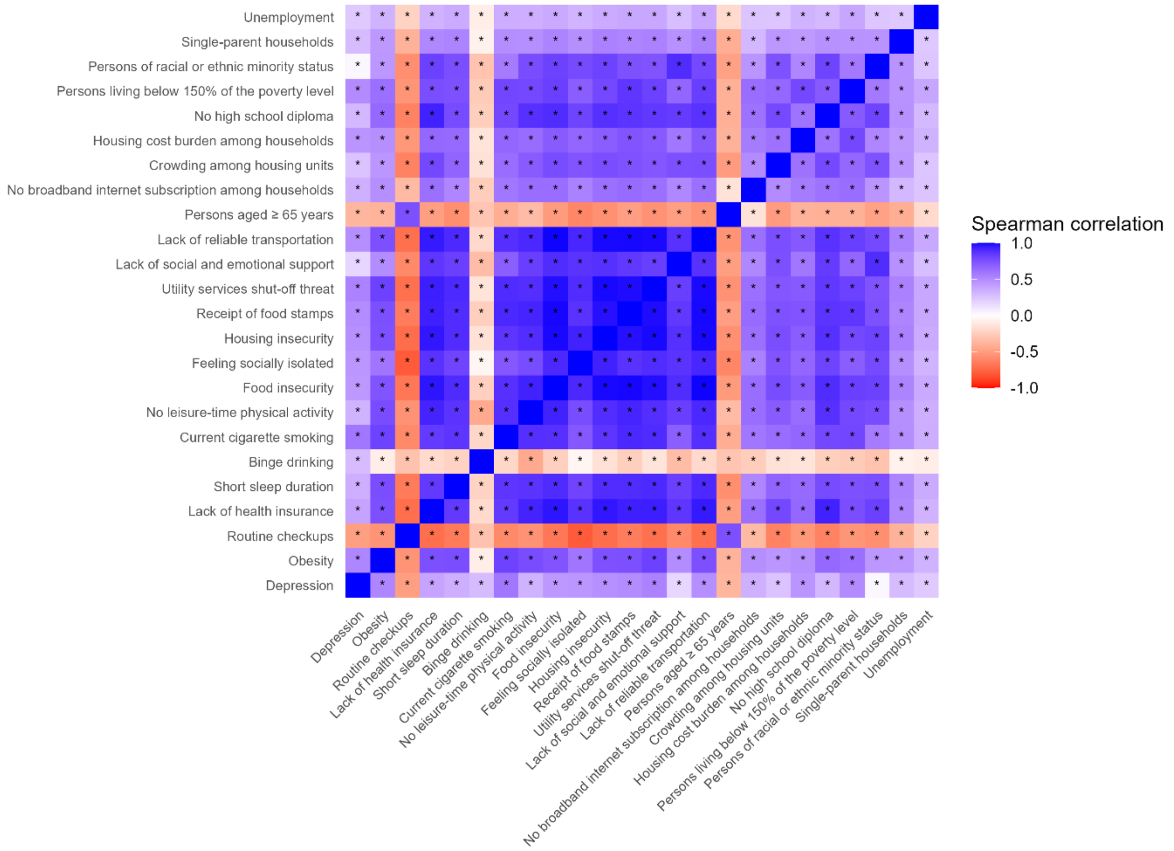

Stars represent statistically significant correlations. Of note, although the correlation coefficients were close to zero, with the lowest and highest being -0.04 and 0.03, none were exactly zero.

© 2025 Descarpentrie A et al. *JAMA Network Open*.

eTable 3. Correlations Between the Considered PLACES Correlates and Diagnosed Diabetes in Southern California (Whole Data Set, n=5420 Census Tracts)

	Spearman Correlations
**Depression**	0.1
**Obesity**	0.57
**Routine checkups**	-0.18
**Lack of health insurance**	0.73
**Short sleep duration**	0.65
**Binge drinking**	-0.7
**Current cigarette smoking**	0.69
**No leisure-time physical activity**	0.88
**Food insecurity**	0.74
**Feeling socially isolated**	0.53
**Housing insecurity**	0.68
**Receipt of food stamps**	0.73
**Utility services shut-off threat**	0.67
**Lack of social and emotional support**	0.7
**Lack of reliable transportation**	0.69
Persons aged ≥ 65 years	-0.03
No broadband internet subscription among households	0.57
Crowding among housing units	0.54
Housing cost burden among households	0.46
No high school diploma	0.74
Persons living below 150% of the poverty level	0.62
Persons of racial or ethnic minority status	0.67
Single-parent households	0.34
Unemployment	0.28

All reported correlations are statistically significant.

Variables modeled using BRFSS (Behavioral Risk Factor Surveillance System) data are shown in bold.

© 2025 Descarpentrie A et al. *JAMA Network Open*.

eFigure 5. Observed vs Estimated (% of Diagnosed Diabetes) Plot in Test Data Set (n=1625 Census Tracts in Southern California); PLACES, 2024

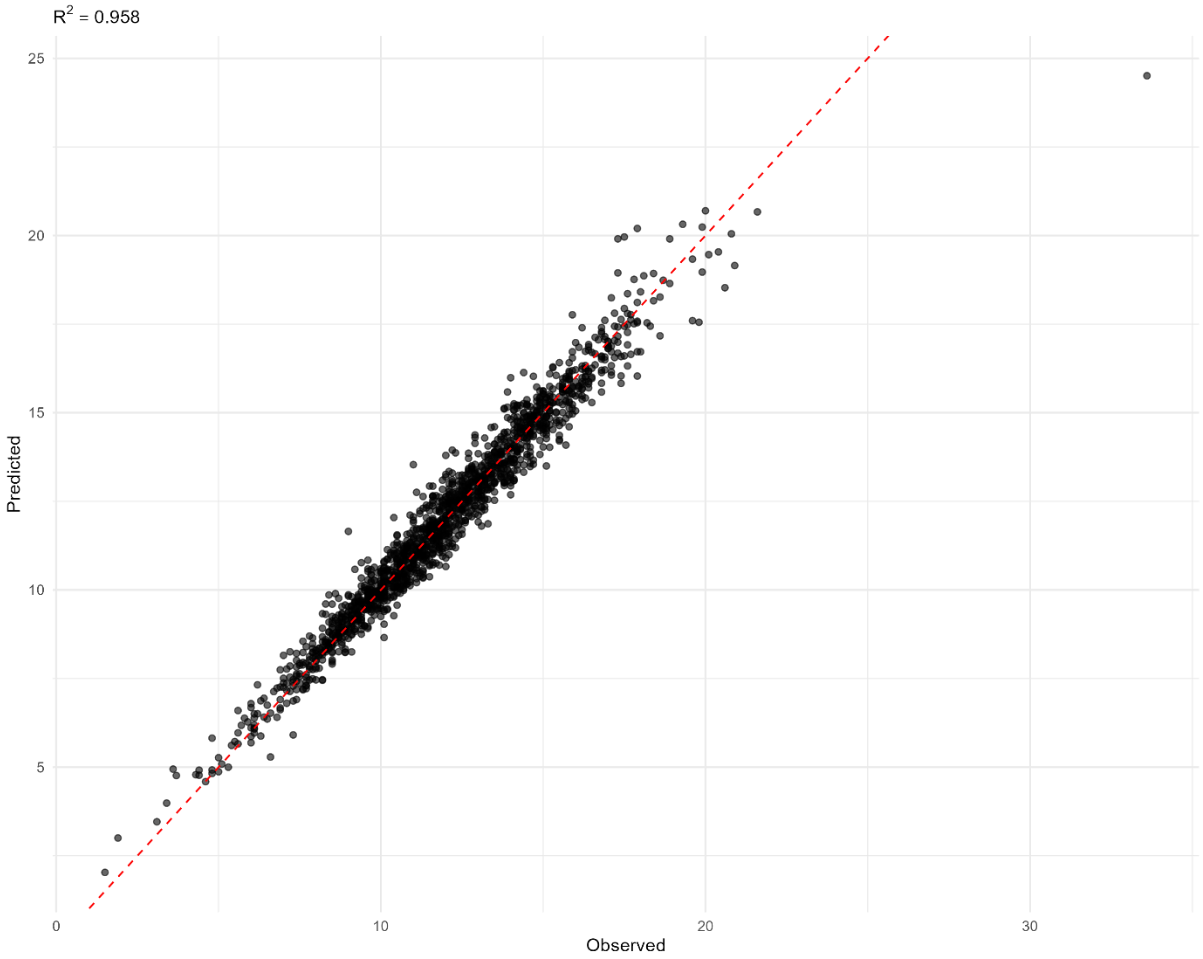

X and y-axis are expressed in % .

© 2025 Descarpentrie A et al. *JAMA Network Open*.

eFigure 6. Correlations Between the Key Correlates of Diagnosed Diabetes in Southern California (Whole Data Set, n=5420 Census Tracts)

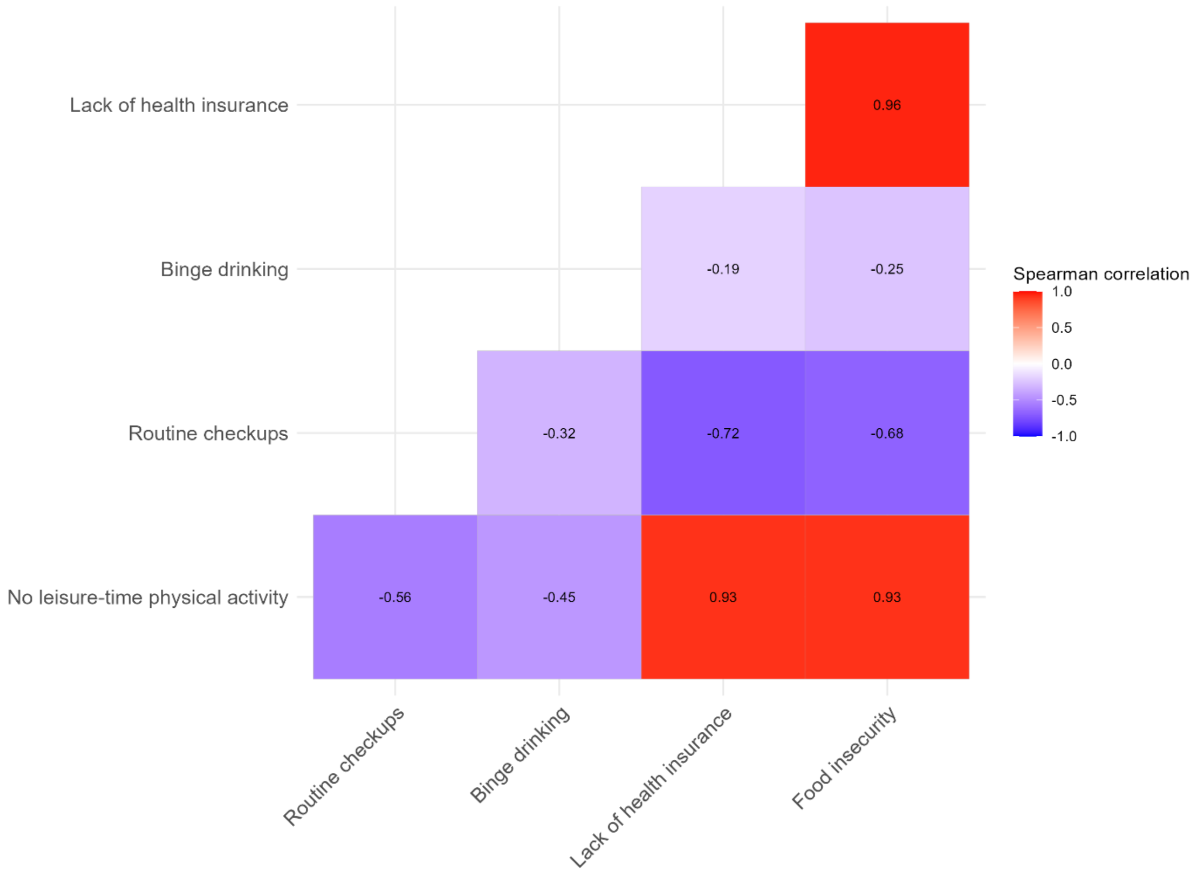

All reported correlations are statistically significant.

© 2025 Descarpentrie A et al. *JAMA Network Open*.

eFigure 7. Correlations (Spearman >0.93) Between the Considered Correlates of Diagnosed Diabetes in Southern California (Whole Data Set, n=5420 Census Tracts)

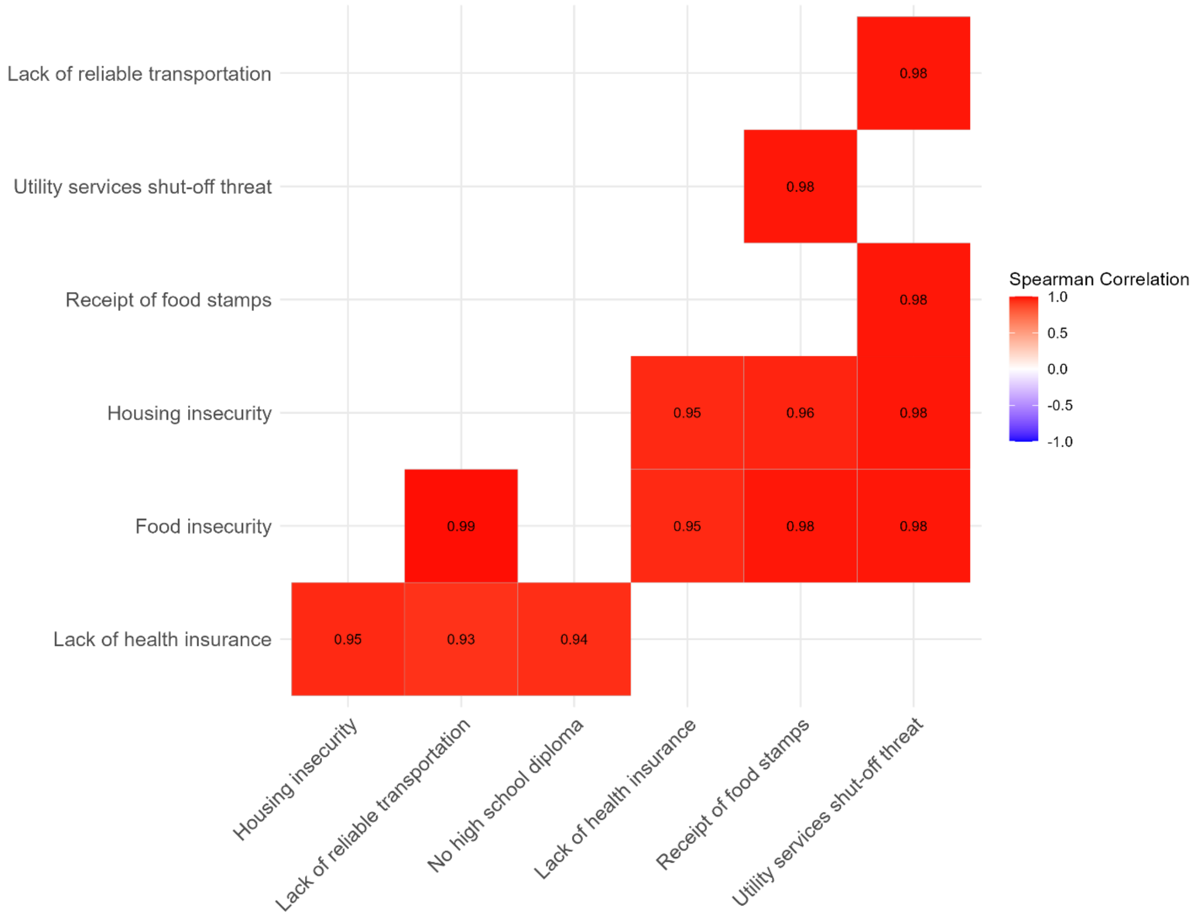

All reported correlations are statistically significant.

© 2025 Descarpentrie A et al. *JAMA Network Open*.

eTable 4. PLACES Variable Characteristics in the US (n= 62 480 Census Tracts)[1]; PLACES, 2024

	Mean of percentages (SE)[1]
**Health Outcomes**	
Depression	22.17 (0.02)
Obesity	33.35 (0.03)
**Prevention**	
Routine checkups	75.55 (0.02)
Lack of health insurance	9.12 (0.02)
**Health-Risk Behaviors**	
Short sleep duration	36.02 (0.02)
Binge drinking	16.54 (0.01)
Current cigarette smoking	13.49 (0.02)
No leisure-time physical activity	23.67 (0.03)
**Health-Related Social Needs**	
Food insecurity	12.92 (0.03)
Feeling socially isolated	33.62 (0.02)
Housing insecurity	11.71 (0.02)
Receipt of food stamps	10.87 (0.03)
Utility services shut-off threat	7.19 (0.02)
Lack of social and emotional support	25.86 (0.02)
Lack of reliable transportation	8.05 (0.01)
**Social Determinants of Health**	
Persons aged ≥ 65 years	13.86 (0.06)
No broadband internet subscription among households	7.74 (0.03)
Crowding among housing units	1.64 (0.01)
Housing cost burden among households	23.86 (0.05)
No high school diploma	7.35 (0.04)
Persons living below 150% of the poverty level	15.87 (0.08)
Persons of racial or ethnic minority status	30.2 (0.18)
Single-parent households	4.22 (0.02)
Unemployment	3.71 (0.02)

[1] standard error

Weighted descriptive statistics (means and standard errors) described the census tracts in the analytic sample, with weights applied to adjust for estimate precision, larger areas had more reliable estimates. Weights were calculated as the inverse of the standard error for each estimate.

© 2025 Descarpentrie A et al. *JAMA Network Open*.

eTable 5. Raw and Normalized Mean Absolute Shapley Additive Explanations Values for Test Dataset With Objective Community-Level Variables Only (n=1625 Census Tracts in Southern California); PLACES, 2024

	Raw mean absolute SHAP value[1]	Normalized mean absolute SHAP value[2]
Persons of racial or ethnic minority status	1.04	0.25
Persons aged ≥ 65 years	0.99	0.24
No high school diploma	0.88	0.21
Persons living below 150% of the poverty level	0.68	0.16
Housing cost burden among households	0.17	0.04
No broadband internet subscription among households	0.15	0.04
Crowding among housing units	0.11	0.03
Single-parent households	0.11	0.03
Unemployment	0.08	0.02

SHAP, Shapley Additive Explanations; [1] expressed in % of diagnosed diabetes prevalence; [2] % of estimated contribution explained

SHAP assigns a unique value to each correlate for every observation (in this case, census tract), indicating how much that variable's value influences the model's estimation relative to the average estimation across the dataset, while accounting for all other variables and interactions. We used normalized mean absolute SHAP values to evaluate each variable’s contribution, identifying a social and behavioral covariate as key if it accounted for at least 5% of the total estimated contribution.

© 2025 Descarpentrie A et al. *JAMA Network Open*.

eTable 6. Raw and Normalized Mean Absolute Shapley Additive Explanations Values for Test Dataset With Individual-Level Variables Only (n=1625 Census Tracts in Southern California); PLACES, 2024

	Raw mean absolute SHAP value[1]	Normalized mean absolute SHAP value[2]
No leisure-time physical activity	1.57	0.3
Routine checkups	0.75	0.14
Lack of health insurance	0.63	0.12
Food insecurity	0.52	0.1
Binge drinking	0.48	0.09
Feeling socially isolated	0.38	0.07
Short sleep duration	0.18	0.03
Obesity	0.16	0.03
Lack of reliable transportation	0.15	0.03
Depression	0.12	0.02
Housing insecurity	0.11	0.02
Receipt of food stamps	0.08	0.01
Current cigarette smoking	0.07	0.01
Lack of social and emotional support	0.07	0.01
Utility services shut-off threat	0.04	0.01

SHAP, Shapley Additive Explanations; [1] expressed in % of diagnosed diabetes prevalence; [2] % of estimated contribution explained

SHAP assigns a unique value to each correlate for every observation (in this case, census tract), indicating how much that variable's value influences the model's estimation relative to the average estimation across the dataset, while accounting for all other variables and interactions. We used normalized mean absolute SHAP values to evaluate each variable’s contribution, identifying a social and behavioral covariate as key if it accounted for at least 5% of the total estimated contribution.

© 2025 Descarpentrie A et al. *JAMA Network Open*.

eTable 7. Raw and Normalized Mean Absolute Shapley Additive Explanations Values For Test Dataset With a Different Splitting Strategy—80/20 (n=1083 Census Tracts in Southern California); PLACES, 2024

	Raw mean absolute SHAP value[1]	Normalized mean absolute SHAP value[2]
No leisure-time physical activity	0.94	0.19
Routine checkups	0.61	0.13
Binge drinking	0.6	0.12
Persons of racial or ethnic minority status	0.4	0.08
No high school diploma	0.29	0.06
Persons aged ≥ 65 years	0.27	0.06
Lack of health insurance	0.27	0.05
Food insecurity	0.23	0.05
Obesity	0.22	0.04
Receipt of food stamps	0.21	0.04
Current cigarette smoking	0.21	0.04
Feeling socially isolated	0.14	0.03
Persons living below 150% of the poverty level	0.07	0.02
Housing cost burden among households	0.07	0.02
Short sleep duration	0.07	0.01
Lack of reliable transportation	0.04	0.01
Housing insecurity	0.04	0.01
Lack of social and emotional support	0.04	0.01
Depression	0.04	0.01
No broadband internet subscription among households	0.03	0.01
Crowding among housing units	0.02	0.01
Single-parent households	0.02	<0.01
Utility services shut-off threat	0.02	<0.01

SHAP, Shapley Additive Explanations; [1] expressed in % of diagnosed diabetes prevalence; [2] % of estimated contribution explained

© 2025 Descarpentrie A et al. *JAMA Network Open*.

SHAP assigns a unique value to each correlate for every observation (in this case, census tract), indicating how much that variable's value influences the model's estimation relative to the average estimation across the dataset, while accounting for all other variables and interactions. We used normalized mean absolute SHAP values to evaluate each variable's contribution, identifying a social and behavioral covariate as key if it accounted for at least 5% of the total estimated contribution.

© 2025 Descarpentrie A et al. *JAMA Network Open*.

eTable 8. Raw and Normalized Mean Absolute Shapley Additive Explanations Values for Test Dataset With a Different Splitting Strategy—75/25 (n=1,354 Census Tracts in Southern California); PLACES, 2024

	Raw mean absolute SHAP value[1]	Normalized mean absolute SHAP value[2]
No leisure-time physical activity	1.31	0.27
Routine checkups	0.68	0.14
Binge drinking	0.52	0.11
Lack of health insurance	0.41	0.09
Persons of racial or ethnic minority status	0.32	0.07
Persons aged ≥ 65 years	0.25	0.05
No high school diploma	0.22	0.05
Current cigarette smoking	0.17	0.04
Food insecurity	0.15	0.03
Obesity	0.14	0.03
Feeling socially isolated	0.13	0.03
Housing cost burden among households	0.07	0.01
Short sleep duration	0.06	0.01
Persons living below 150% of the poverty level	0.06	0.01
Housing insecurity	0.06	0.01
Lack of reliable transportation	0.05	0.01
Lack of social and emotional support	0.04	0.01
Receipt of food stamps	0.04	0.01
Depression	0.04	0.01
Utility services shut-off threat	0.03	0.01
Crowding among housing units	0.03	0.01
Single-parent households	0.02	<0.01
No broadband internet subscription among households	0.01	<0.01

SHAP, Shapley Additive Explanations; [1] expressed in % of diagnosed diabetes prevalence; [2] % of estimated contribution explained

© 2025 Descarpentrie A et al. *JAMA Network Open*.

SHAP assigns a unique value to each correlate for every observation (in this case, census tract), indicating how much that variable's value influences the model's estimation relative to the average estimation across the dataset, while accounting for all other variables and interactions. We used normalized mean absolute SHAP values to evaluate each variable's contribution, identifying a social and behavioral covariate as key if it accounted for at least 5% of the total estimated contribution.

© 2025 Descarpentrie A et al. *JAMA Network Open*.

eTable 9. Raw and Normalized Mean Absolute Shapley Additive Explanations Values for Test Dataset, Taking Into Account Uncertainty in Analyzed Variables (n=1625 Census Tracts in Southern California); PLACES, 2024

	**Raw mean absolute SHAP value[1] [95% CI]**	**Normalized mean absolute SHAP value[2]**
No leisure-time physical activity	1.36 [1.36;1.37]	0.27
Binge drinking	0.51 [0.49;0.51]	0.1
Routine checkups	0.5 [0.5;0.51]	0.1
Food insecurity	0.38 [0.32;0.38]	0.07
Persons aged ≥ 65 years	0.3 [0.3;0.31]	0.06
Lack of health insurance	0.29 [0.26;0.29]	0.06
Persons of racial or ethnic minority status	0.2 [0.19;0.2]	0.04
Current cigarette smoking	0.19 [0.19;0.19]	0.04
Lack of reliable transportation	0.18 [0.18;0.19]	0.03
Receipt of food stamps	0.15 [0.14;0.19]	0.03
No high school diploma	0.13 [0.13;0.16]	0.03
Obesity	0.13 [0.12;0.13]	0.02
Housing insecurity	0.11 [0.11;0.12]	0.02
Feeling socially isolated	0.1 [0.1;0.1]	0.02
Depression	0.1 [0.08;0.1]	0.02
Housing cost burden among households	0.09 [0.09;0.1]	0.02
Crowding among housing units	0.07 [0.03;0.07]	0.01
Short sleep duration	0.06 [0.06;0.06]	0.01
Persons living below 150% of the poverty level	0.06 [0.06;0.07]	0.01
Utility services shut-off threat	0.05 [0.04;0.05]	0.01
No broadband internet subscription among households	0.05 [0.05;0.05]	0.01
Single-parent households	0.05 [0.03;0.05]	0.01
Lack of social and emotional support	0.03 [0.03;0.05]	0.01
Unemployment	0.03 [0.03;0.03]	0.01

© 2025 Descarpentrie A et al. *JAMA Network Open*.

CI, confidence interval (from bootstrapping procedure); SHAP, Shapley Additive Explanations; [1] expressed in % of diagnosed diabetes prevalence; [2] % of estimated contribution explained

SHAP assigns a unique value to each correlate for every observation (in this case, census tract), indicating how much that variable's value influences the model's estimation relative to the average estimation across the dataset, while accounting for all other variables and interactions. We used normalized mean absolute SHAP values to evaluate each variable’s contribution, identifying a social and behavioral covariate as key if it accounted for at least 5% of the total estimated contribution.

© 2025 Descarpentrie A et al. *JAMA Network Open*.

eTable 10. Raw and Normalized Mean Absolute Shapley Additive Explanations Values for Test Dataset, Using K-Fold (k=10) Cross-Validation (n=1625 Census Tracts in Southern California); PLACES, 2024

	Raw mean absolute SHAP value[1]	Normalized mean absolute SHAP value[2]
No leisure-time physical activity	1.49	0.31
Routine checkups	0.67	0.14
Binge drinking	0.51	0.11
Lack of health insurance	0.32	0.07
Food insecurity	0.27	0.06
Current cigarette smoking	0.21	0.04
Persons of racial or ethnic minority status	0.21	0.04
Persons aged ≥ 65 years	0.19	0.04
Obesity	0.12	0.02
Receipt of food stamps	0.11	0.02
Short sleep duration	0.1	0.02
Housing insecurity	0.09	0.02
Feeling socially isolated	0.08	0.02
No high school diploma	0.08	0.02
Lack of reliable transportation	0.08	0.02
Depression	0.07	0.01
Persons living below 150% of the poverty level	0.07	0.01
Housing cost burden among households	0.06	0.01
Lack of social and emotional support	0.06	0.01
Crowding among housing units	0.02	<0.01
Utility services shut-off threat	0.02	<0.01
Single-parent households	0.01	<0.01
No broadband internet subscription among households	0.01	<0.01
Unemployment	0.01	<0.01

SHAP, Shapley Additive Explanations; [1] expressed in % of diagnosed diabetes prevalence; [2] % of estimated contribution explained

© 2025 Descarpentrie A et al. *JAMA Network Open*.

SHAP assigns a unique value to each correlate for every observation (in this case, census tract), indicating how much that variable's value influences the model's estimation relative to the average estimation across the dataset, while accounting for all other variables and interactions. We used normalized mean absolute SHAP values to evaluate each variable's contribution, identifying a social and behavioral covariate as key if it accounted for at least 5% of the total estimated contribution.

© 2025 Descarpentrie A et al. *JAMA Network Open*.

eReferences

1. Mezuk B, Eaton WW, Albrecht S, Golden SH. Depression and Type 2 Diabetes Over the Lifespan. *Diabetes Care*. 2008;31(12):2383-2390. doi:10.2337/dc08-0985

2. Klein S, Gastaldelli A, Yki-Järvinen H, Scherer PE. Why does obesity cause diabetes? *Cell Metabolism*. 2022;34(1):11-20. doi:10.1016/j.cmet.2021.12.012

© 2025 Descarpentrie A et al. *JAMA Network Open*.
