# Supplementary material for: Social and Behavioral Factors Associated With Diabetes in Southern California vs the US
Source: JAMA Netw Open. 2025 Oct 22;8(10):e2538377. doi: 10.1001/jamanetworkopen.2025.38377 (PMC12547587; doi:10.1001/jamanetworkopen.2025.38377)
Supplement: Supplement 2. — Data Sharing Statement [file jamanetwopen-e2538377-s002.pdf]

# Data Sharing Statement

Descarpentrie. Social and Behavioral Correlates of Diabetes in Southern California vs the United States. *JAMA Netw Open*. Published October 20, 2025.
doi:10.1001/jamanetworkopen.2025.38377

**Data**
**Data available:** Yes
**Data types:** Data (not involving human participants)
**How to access data:** The combined datasets used and/or analyzed during the current study are available from the corresponding author on reasonable request. Individually, each of the datasets used in this study are publicly available through this website: a) https://data.cdc.gov/500-Cities-Places/PLACES-Local-Data-for-Better-Health-Census-Tract-D/cwsq-ngmh/about_data b) https://atsdr.cdc.gov/place-health/php/svi/svi-data-documentation-download.html
**When available:** With publication

**Supporting Documents**
**Document types:** None

**Additional Information**
**Who can access the data:** The combined datasets used and/or analyzed during the current study are available from the corresponding author on reasonable request. Individually, each of the datasets used in this study are publicly available through this website: a) https://data.cdc.gov/500-Cities-Places/PLACES-Local-Data-for-Better-Health-Census-Tract-D/cwsq-ngmh/about_data b) https://atsdr.cdc.gov/place-health/php/svi/svi-data-documentation-download.html
**Types of analyses:** All
**Mechanisms of data availability:** With investigator support
